# Effectiveness of planned teaching intervention on knowledge and practice of breast self-examination among first year midwifery students

**DOI:** 10.1371/journal.pone.0184636

**Published:** 2017-09-26

**Authors:** Hiwot Abera, Daniel Mengistu, Asres Bedaso

**Affiliations:** 1 Hawassa University, College of Medicine and Health Sciences, School of Nursing and Midwifery, Hawassa, Ethiopia; 2 Addis Ababa University, College of Medicine and Health Sciences, Department of Nursing and Midwifery, Addis Ababa, Ethiopia; School of Health, Ordu University, TURKEY

## Abstract

**Introduction:**

The prevalence of cancer is growing rapidly in all parts of the word and Ethiopia is no exception. Secondary prevention, as simple as monthly breast self-examination, is the best option to tackle the rising of this epidemic. Health awareness programs on screening and early detection are the corner stones to reduce the morbidity and mortality resulting from breast cancer.

**Objective:**

The aim of the study is to assess the effectiveness of planned teaching program on knowledge and practice of breast self-examination among first year female midwifery students in Hawassa health Sciences College.

**Methods and materials:**

A pre-experimental one group pre-posttest design was used among 61 students who were selected by systematic random sampling technique. Data was collected using structured questionnaire and adapted and approved checklist. Data was entered using Epi-Info and analyzed using SPSS version 20. Pre-and post-intervention results were calculated using paired t-test.

**Results:**

The mean age of the study participants was 20.13(±2.27) and 77% of the study participants were single. Before the intervention 14(23%) of respondents had information and practiced breast self-examination, only 8(13.1%) performed breast self -examination on a regular monthly basis. The number and percentage of the knowledgeable respondents pre-post intervention is 23(37.7%) and 35(57.4%), respectively. The mean knowledge difference for the pre-post intervention is 0.18±0.695 (P < 0.05). The respondents’ pre- post interventions score of satisfactory practical competency were 10(16.4%) and 43(70.5%), respectively as well. The mean net gain for the pre-post breast self-examination intervention is 0.51± 0.62 (P < 0.001). Both the knowledge and practical competency scores showed highly significant increment after the intervention, showing that the research hypothesis was accepted.

**Conclusion and recommendations:**

Planned teaching intervention on knowledge and Breast self-examination of students has resulted in an increment of both knowledge and the practice of breast self-examination. Teaching breast self-examination with demonstration to all at risk groups as a secondary prevention for breast cancer and large scale studies on heterogeneous groups is important.

## Introduction

Breast cancer is the most common cancer seen in women, constituting 22% of all cancer cases worldwide [1–3]. Although there is no cancer registry in Ethiopia, hospital records show that there are more than 200,000 cancer cases per year where cervical and breast cancers are the top two cancer types [4].

Breast cancer is a malignant proliferation of epithelial cells lining the ducts or lobules of the breast. In the year 2004, about 216,000 cases of invasive breast cancer and 40,000 deaths occurred in the United States alone. Women who experience menarche at age 16 have only 50 to 60% of the breast cancer risk of a woman having menarche at age 12; the lower risk persists throughout life [5].

At present, despite the magnitude of the problem, no practical method of primary preventions available. However, an advanced stage of breast cancer is clearly associated with a poor prognosis. Thus, screening (secondary prevention), which aims at detecting and treating the disease at early stage before it metastasizes, is an important strategy for the control of this disease. In the absence of public education programs on breast cancer, detection often occurs at late stage, especially in the developing countries [6]. Yet a lower incidence of advanced breast cancer or death from breast cancer among women who were carefully instructed in the methods of Breast Self-Examination (BSE) and adequately implemented the program using optimal visual and palpatory techniques is achieved [7].

Several studies, based on breast cancer patients' retrospective self-report on their practice of the breast self- exam, have established that a positive association exists between performance of the exam and early detection of breast cancer. There is also evidence that most of the early breast tumors are self-discovered, and that most early self-discoveries are by BSE performers. Moreover, the need for greater understanding of the social-psychological factors affecting acceptance of BSE are of growing concern [8].

Even though studies show that breast self-examination can contribute to the early detection of non-invasive breast cancer and as an easy and private detection method, there exists a knowledge and practice gap especially in developing countries which is elicited by the increasing number of cancer morbidity and mortality.

## Methods and materials

### Study design and setting

A pre-experimental study with one group pre-and posttest design was conducted to assess Effectiveness of Planned Teaching Intervention on Knowledge and Practice of Breast Self-Examination among First Year Female Midwifery Students.

The study was conducted from October 2014 to May 2015 in Hawassa Health Sciences College, Southern Nations, Nationalities and Peoples Region, Hawassa, Ethiopia. Hawassa city is located 280 km South of Addis Ababa and the capital city of the Southern Nations, Nationalities and Peoples Region (SNNPR). Hawassa Health Science College was established in 1979 and runs diploma training programs in Health Sciences.

### Sample size determination and sampling procedures

The study population were female midwifery year I students. Systematic random sampling method was used to select all 64 female subjects included in the study from a total of 192 students.

### Data collection instrument

A standardized questionnaire consisting of three tools such as socio-demographic characteristics, knowledge and practice (skill) assessment was used to collect the data. The instrument was developed based on the literature survey and approved for their validity by experts in the area—The instrument was initially prepared in English and then translated in to Amharic Language by language experts and retranslated back to the English Language. The translation and retranslation were found to be the same.

The designed teaching package was validated by experts, which included theoretical (module) and practical session like lecturing, audiovisuals and hand- on demonstration, focused on BSE knowledge and practice. An observer checklist was prepared containing all the necessary steps to measure the performance (skill) before and after the intervention for practice measurement. Data were collected by trained data collectors first one week before the intervention then the post test was done after 45 days of the intervention.

### Data processing and analysis

The collected data was checked for completeness and consistency on daily basis by the immediate supervisor and then manually edited and coded to ensure data quality. Then data was entered into a computer and analyzed using Epi-Info 3.5.1 and SPSS version 20 software respectively.

Variables were recorded and initially descriptive statistics was calculated. Means and standard deviations were calculated for continuous variables while proportions and frequencies for categorical variables. The paired t-test was used to compare the changes in scores from pre- to post intervention and to assure the net gain. Alpha level of p < 0.05 was considered to be statistically significant. Tables, graphs, charts and texts were used for data presentation.

### Ethics statement

Ethical clearance was obtained from the Institutional Care and Use Committee (IACUC) of Nursing and Midwifery, College of Health Sciences, Addis Ababa University. A formal letter was written to Department of Midwifery, Hawassa College of Health Sciences for permission to carry out the study. Verbal and written consents were obtained from the study subjects after explaining the objectives, benefits and risks of the study. Individuals in the study group who were found to have -suspicious findings during the study were sent to clinicians for further investigation and appropriate management. Privacy and confidentiality were maintained. The subjects were told that any information they provided would remain confidential. Their names never appeared on the data collection instrument and- they were also informed that their responses would be used only for the purpose of the study.

## Result

The total number of participants was 61 with 100% response rate. The mean age of the study participants was 20.13(± 2.27), 47(77%) of the study participants were single and majority 38 (62.3%) of the students were protestant by religion and the average monthly family income of the participants was 1622.54 ETB “S1 Table”.

### Knowledge of BSE

From the total study participants 14(23%), 61(100%) have heard of BSE in the pre intervention and post intervention phase consecutively “Table 1”.

**Table 1:** Knowledge score on breast self-examination among respondents before and after the intervention, in Hawassa Health Science College, 2015 (N = 61).

**Variable**	**Pre-intervention**	**Post- intervention**
**Knowledge**	Yes No	Yes No
	N (%) N (%)	N (%) N (%)
Heard of BSE	14(23.0) 47(77)	61(100.0) 0(0.0)
Steps to follow during BSE	19(31.1) 42(68.9)	60(98.4) 1(1.6)
Identify breast mass by looking in the mirror	24(39.3) 37(60.7)	60(98.4) 1(1.6)
Monthly BSE helps early detection of lump	20(32.8) 41(67.2)	59(96.7) 2(3.3)
Usage of correct part of the finger to examine	20(32.8) 41(67.2)	59(96.7) 2(3.3)
Susceptibility to breast cancer	20(32.8) 41(67.2)	52(85.2) 9(14.8)
Benefit of BSE	30(49.2) 31(50.8)	50(82.0) 11(18)
Barriers towards BSE	20(32.8) 41(67.2)	47(77.0) 14(23)
Seriousness of breast cancer	25(41.0) 36(59.0)	48(78.7) 13(21.3)

### Practical competency of BSE

All values of the observers check list show significant increment from nil to high scores except components like observation through the mirror 9(14.8%), squeezing the nipple 2(3.3%) and looking for symmetry and dimpling 1(1.6%) which had values different from 0 before the intervention.

The competency check lists in pre intervention period showed that 9(14.8%) of the respondents reported observation through the mirror while checking for breast cancer, this figure rise to 57(93.4%) after intervention. 2(3.3%)of the respondents reported for squeezing the nipples as a component of examination during pre-intervention and the figure rise to 53(86.9%) after intervention. 1(1.6%) observed for symmetry of breast dimpling before the intervention and this figure increased to 50(82%) after the intervention.

The other components of Breast self-examination were found to be unnoticed and unpracticed. Post intervention values indicated an increment in all values of the observation check list. 47(77%) of the respondents were able to find multiple lumps from silicone breast model simulation “Table 2”.

**Table 2:** Distribution of the practical competency of BSE among respondents in Hawassa Health Sciences College, 2015 (N = 61).

Variable	Pre-intervention		Post- intervention		
Practice	S	NS	S	NS	
	N (%)	N (%)	N (%)	N (%)	
Observes breasts through mirror	9(14.8)	52(85.2)	57(93.4)		4(6.6)
Maintains correct position during BSE	0(0.0)	61(100)	58(95.1)		3(4.9)
Examines all breast areas	0(0.0)	61(100)	53(86.9)		8(13.1)
Applies adequate pressure	0(0.0)	61(100)	57(93.4)		4(6.6)
Uses circular motion with each type of pressure	0(0.0)	61(100)	50(82)		11(18)
Use of pads of the three middle fingers	0(0.0)	61(100)	59(96.7)		2(3.3)
Uses of the vertical Strip/circular pattern	0(0.0)	61(100)	60(98.4)		1(1.6)
Squeezes nipples to check for discharge	2(3.3)	59(96.7)	53(86.9)		8(13.1)
Examines breasts Symmetric dimpling/retraction	1(1.6)	60(98.4)	50(82)		11(18)
Detects lumps in breast	0(0.0)	61(100)	47(77)		14(23)

S: Satisfactory NS: Not satisfactory

**Fig 1:**
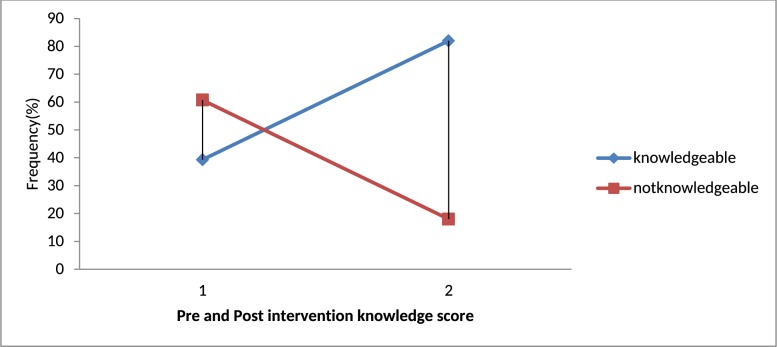
Pre- and post-interventions knowledge level of respondents in Hawassa Health Sciences College, 2015.

**Fig 2:**
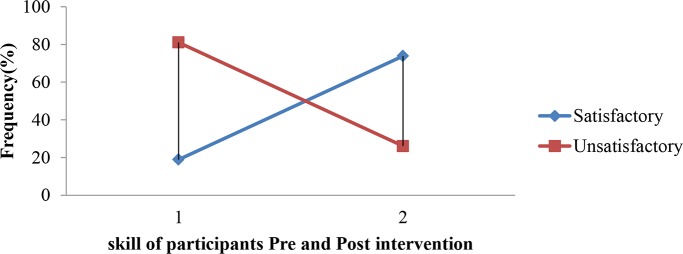
Pre- and post- interventions skill level of respondents in Hawassa Health Sciences College, 2015.

The level of knowledge obtained after intervention fell in to satisfactory group which was detected by increment from 23(37.7%) to 35(57.4%). The same is true regarding practical competency which increased from 10(16.4%) to 43(70.5%) before and after the intervention, respectively “Figs 1 and 2.” respectively.

The mean difference (net gain) in knowledge and practical competency scores were computed using paired t-test. The CI and P values showed highly significant associations in both knowledge and skill assessments with P < 0.05 each. This explains that, the mean difference doesn’t come by chance but is the result of the intervention introduced “Table 3”.

**Table 3:** Comparison of mean differences on knowledge and practical competency before and after the intervention in Hawassa Health Science College, 2015 (N = 61).

**Variable**	**Mean difference (SD)**	**t test**	**d.f**	**CI**	**P value**
Knowledge score	0.46± 0.64	5.166	60	(0.26–0.59)	0.00**
Practice competency	0.51± 0.62	6.803	60	(0.38–0.70)	0.00**

** Significant at P< 0.05

## Discussion

At base line, the knowledge score of this study revealed that, 38(62.3%) of the respondents were not knowledgeable. This finding is in line with a study done in Western New York [8], in which lower score is obtained on Knowledge index. Moreover, in a study done by in Nigeria [9] less than 46.8% of study group and 45.7% of control group had good knowledge before the intervention and one quarter of participants were categorized as having satisfactory knowledge in similar study in Legos [10].

In a study done in Southeastern Iran [11] only 21.6% of women had good knowledge about BSE. In contrary to the finding of Southeastern Iranian study, results from female health care workers in Tehran 63% of them know about BSE and in Turkey [12] more than half of the nursing and midwifery students had sufficient information about BSE. In Ilorin Nigeria [13] 95.6% of respondents were aware of BSE, 66.5% of Sudanese students know about BSE [9]. This difference in baseline values may be the Education and Health policy focus of the countries towards preventive behaviors.

Before any intervention, the practical competency of most of the participants is not satisfactory. In this study, the satisfactory result for pre-intervention practical competency was 10(16.4%). In line with this finding, a study done in Southeastern Iran [11] only 4.6% of women in Zehdan perform BSE and 6% of women in Tehran performed BSE in a regular basis, Iran [14] respectively. Similarly, 1% of students in Makerere University in Uganda accurately demonstrated BSE [15], 54.8% among female secondary school teachers in Ilorin Nigeria [13], 19.0% of female undergraduate students in Northwest Nigeria [2] perform BSE monthly and 11%practice it regularly among Legos secondary teachers [10]. The study done on Sudanese students [9] showed a result which is in line with this study result, which is 7.2% of them used to practice BSE. In contrary, in study done- among women living in one urban and two semi-urban Aegean countries [16], BSE showed higher rate than other screening methods which is 61.7% this difference may be the promotion of BSE in these countries unlike our country.

Specific techniques of BSE are limited before the intervention. For instance, in this study no one demonstrated correct position of BSE. In line with this result, a study which is done among Turkish [12] Nursing and Midwifery students by, showed only one fifth of the respondents used recommended BSE position and techniques and in Sudan 41(20.5%) of medical students showed the correct positions of BSE [9]. In this study, only 2(1.6%) showed the skill of squeezing the nipple. Similarly, in study done on Sudanese Medical students [9], 98(49%) showed squeezing the nipple before the intervention.

Interventions towards enhancing BSE are found to be effective ways of increasing knowledge and practical competency of BSE. This study revealed that use of combination methods of instruction namely lecture, audiovisuals and demonstration as effective ways of delivering BSE information and skill. On a community based interventional study done by in Pakistan, Karachi [17] leaflet and tape/slide programs in combination were found effective in increasing knowledge of BSE. In addition, the finding of the study done on Sudanese Medical students [9] was consistent with the finding of this study. This is, by using teaching module, comprising lecture/discussion, video presentation, demonstration, clinical teaching on breast models and use of IEC materials improvement in BSE knowledge was seen.

After the intervention, we expect a change from the base line data. In this study, except socio demographic characters, all the values show a significant increment in posttest analysis. This study revealed that the knowledge score rose to 50(82.0%), P = 0.000. Similarly, a study done in Rural Women in India [18], showed a significant increase in knowledge score (z = -15.807; P<0.001), and a study done in Pakistan [19] also showed the knowledge score rising to 83% (P< 0.001).

The practical competency score rise to satisfactory after teaching intervention was given. In this study, the posttest practical competency score was satisfactory for 43(70.5%).This is consistent with other study done in Pakistan [19] in which 38(54%) practice BSE after intervention showing a significant increment (P< 0.001). Under a randomized educational intervention in India, performance of BSE 321/ 342 (93%) was observed showing significant increment from the baseline value. A study done in Nigeria [20] also supported the finding of this study, by showing an increment from 57.7% to 76.0% in the proportion of the respondents that had practiced breast self-examination in the study group after the health education. The result of longitudinal study in Sudan among Medical students [9] is also in line with this study, the practice value of Medical students rose to 73.9% adhering on regular monthly BSE after the teaching intervention.

Specific techniques of BSE also showed progress after the intervention. This study revealed those who score satisfactory towards correct position of BSE are 58(95.1%). In line with this, a study done in Sudanese Medical students [9] showed increment to 174(73.5%) on score of correct position of BSE. Squeezing nipples as a technique of BSE is found to increase to 53(86.9%) after the intervention in this study. Similarly, a study done in Sudan [9], showed-increment after intervention to 127(63.5%).

The pretest and posttest differences showed a significant improvement on Knowledge and Practice due to the intervention introduced. In this study, the mean scores of knowledge showed a significant difference from baseline value by t (60) = 5.16, CI (0.261–0.591), P = 0.00.This is consistent with a study done in Sudan (X^2^ = 91.672, d.f = 5, P = 0.000) [9]. Similar improvement was seen in a study done on female students of the tertiary institutions in a Nigerian state [20] following the health education intervention. There was remarkable improvement in the level of knowledge of respondents in the study group on breast cancer and Breast Self- Examination (P = 0.00). On study done among Egyptian working women [21] after program implementation a notable difference in participants’ level of knowledge, attitude and practice was observed. The differences were statistically highly significant (P = <0.01). Similarly, in study done in Pakistan Karachi [19] both the knowledge and Practice of BSE show difference which is highly significant (p<0.001).

## Conclusion and recommendations

Based on the study findings, planned teaching on BSE was found to be effective in improving the knowledge and practical skills of female students. The methods used for training the students about BSE such as lecture/discussion, video demonstrations and hands on practicum were found to be effective ways of delivering BSE knowledge and skill.

As a secondary prevention of breast cancer, nurses should teach about BSE by using demonstration and supervision to improve the knowledge and practical competency of females at risk for breast cancer.

School and other institutions should teach about BSE targeting at risk groups as a secondary prevention of breast cancer. Also, it will be better if schools try to incorporate BSE teachings in their curricula.

## Supporting information

S1 Table Socio demographic characteristics of study subjects, in Hawassa Health Science College, 2015 (N = 61). (DOCX)
