# Supplementary material for: Effectiveness of planned teaching intervention on knowledge and practice of breast self-examination among first year midwifery students
Source: PLoS One. 2017 Sep 26;12(9):e0184636. doi: 10.1371/journal.pone.0184636 (PMC5614528; doi:10.1371/journal.pone.0184636)
Supplement: S1 Table — (DOCX) [file pone.0184636.s001.docx]

**S1 Table. Socio demographic characteristics of study subjects, in Hawassa Health Science College, 2015 (N =61)**

| Variables | Number (%) |
| --- | --- |
| Age 18-23 | 56(91.8) |
| 24-28 | 5(8.2) |
| Total | 61(100) |
| Marital Status Married | 14(23) |
| Single | 47(77) |
| Total | 61(100) |
| Religion Orthodox | 11(18 |
| Protestant | 38(62.3) |
| Muslim | 8(13.1) |
| Catholic | 2(3.3) |
| Others | 2(3.3) |
| Total | 61(100.0) |
| Family Monthly income in ETB |  |
| <500 | 27(44.3) |
| >500 | 34(55.7) |
| Total | 61(100.0) |

ETB: Ethiopian Birr (1ETB=0 .056$)
